# Effect of Surfactants on the Splashing Dynamics of Drops Impacting Smooth Substrates

**DOI:** 10.1021/acs.langmuir.3c03248

**Published:** 2024-03-06

**Authors:** Nonu Varghese, Thomas C. Sykes, Miguel A. Quetzeri-Santiago, Alfonso A. Castrejón-Pita, J. Rafael Castrejón-Pita

**Affiliations:** † School of Engineering and Material Sciences, Queen Mary University of London, London, E1 4NS, U.K.; ‡ Department of Mechanical Engineering, University College London, Torrington Place, London WC1E 7JE, U.K.; § Department of Engineering Science, University of Oxford, Oxford OX1 3PJ, U.K.; ∥ Instituto de Investigaciones en Materiales, Universidad Nacional Autónoma de México, Cd. Universitaria, Mexico City 04530, Mexico

## Abstract

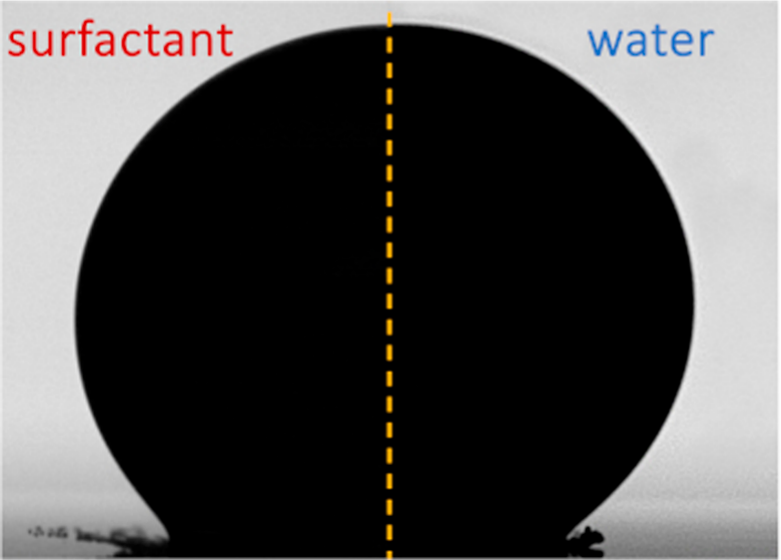

We present the results of a systematic study elucidating the role that dynamic surface tension has on the spreading and splashing dynamics of surfactant-laden droplets during the impact on hydrophobic substrates. Using four different surfactants at various concentrations, we generated a range of solutions whose dynamic surface tension were characterized to submillisecond timescales using maximum bubble-pressure tensiometry. Impact dynamics of these solutions were observed by high-speed imaging with subsequent quantitative image processing to determine the impact parameters (droplet size and speed) and dynamic wetting properties (dynamic contact angle). Droplets were slowly formed by dripping to allow the surfactants to achieve equilibrium at the free surface prior to impact. Our results indicate that while only the fastest surfactants appreciably affect the maximum spreading diameter, the droplet morphology during the initial stages of spreading is different to water for all surfactant solutions studied. Moreover, we show that surfactant-laden droplets splash more easily than pure liquid (water). Based on the association of the splashing ratio to our tensiometry measurements, we are able to predict the effective surface tension acting during splashing. These results suggest that droplet splashing characteristics are primarily defined by the stretching of the equilibrated droplet free surface.

## Introduction

Understanding droplet dynamics and wetting is crucial in many industrial processes such as inkjet, 3D printing, coating, and crop spraying.^1−4^ The study of the droplet impact was pioneered by Worthington at the end of the 19th century.^5^ Since then, much research has been dedicated to reveal the physics of the droplet impact, with many reviews available in the scientific literature.^6,7^ Impact outcomes depend on the liquid and substrate properties, and the ambient gas density and viscosity.^8−12^ In brief, a droplet impacting on a flat solid substrate can splash, or not, depending on the impact characteristics.^13,14^ Recent work has also demonstrated that the nanoparticles play a crucial role in the postimpact dynamics, including modifying the splashing threshold.^4,15^ Most industrial applications desire to operate in no-splashing conditions, e.g., the quality of inkjet printing relies on splash-free smooth deposition of ink droplets on a solid substrate. In crop spraying, over 50% of pesticides applied can be wasted as they bounce and splash, dispersing into the soil and atmosphere.^16^ In this paper, our primary focus is on understanding the spreading and splashing dynamics of surfactant-laden droplets.

The splashing behavior of liquid droplets impacting solid substrates is usually presented in terms of various dimensionless parameters such as the Weber number, *We* = ρ*D*_0_*U*_0_^2^/σ, and the Reynolds number, *Re* = ρ*D*_0_*U*_0_/μ, where *U*_0_ is the impact velocity, *D*_0_ is the diameter, and ρ, μ, and σ are the droplet density, dynamic viscosity, and surface tension, respectively.^8,17^ However, these parameters do not account for the crucial role of the surrounding gas on the dynamics of the lamella,^10^ or wettability.^14^ In 2014, Riboux and Gordillo combined the potential flow theory, the momentum balance equation, and aerodynamic lubrication at the lifting of the lamella to develop a model in which the resulting ejection velocity is balanced by capillary retraction. This model established a splashing threshold through a parameter known as the splashing ratio β, showing good agreement with experiments.^18^ For low-viscosity liquids (Oh = *We*^1/2^/*Re*≪1) at atmospheric pressure, the splashing ratio is given by
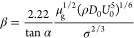
1where μ_g_ is the gas viscosity and α is the angle between the lifted lamella and the substrate at the onset of splashing, which is found to be usually 60°.^19^ The dimensionless number, defined as the splashing ratio β, indicates the magnitude of the aerodynamic forces needed to overcome the surface tension to break up the liquid sheet into smaller droplets.^18,19^ Together with the maximum advancing contact angle θ_max_, the splashing ratio has recently been used to parametrize the splashing behavior of droplets impacting smooth hydrophilic, hydrophobic, superhydrophobic,^14^ rough,^12^ and curved substrates.^20^

Most natural and industrial processes involve droplets containing impurities, added either intentionally or unintentionally, including colloids, particles, and polymers. Surfactants are among the most common additives in industry, since their amphiphilic nature causes them to adsorb at free surfaces, enabling surface tension and interfacial properties to be modified.^21^ Surfactant dynamics at liquid interfaces are complicated and the focus of many recent studies.^22−24^ For example, it has been shown that during the jetting and formation of droplets, surfactants remain at the droplet front to then diffuse to the rest of the newly formed surface.^22^ It is generally accepted that the surface tension of a *freshly formed* free surface in a surfactant solution has an initial surface tension close to that of the solvent σ_0_, with surfactants contained in the bulk. As surfactants diffuse to, and then adsorb at, the free surface, the surface tension decreases due to the adsorbed surfactant monolayer disrupting the cohesive forces between the solvent molecules. When the adsorbing and desorbing flux of individual surfactants is equal, the free surface attains its equilibrium value σ_∞_. Surfactant solutions therefore exhibit a *dynamic* surface tension σ(*t*) arising from the transient process of diffusion and adsorption, where σ ≈ σ_0_ at *t* ≈ 0 and σ → σ_∞_ as *t* → ∞. At low concentrations, surfactants exist as individual molecules in the bulk, with σ_∞_ decreasing as the surfactant concentration increases. However, above the *critical micelle concentration* (CMC), most “excess” surfactants form aggregate structures (e.g., micelles, vesicles, and bilayers), with the hydrophobic part of the amphipathic surfactants being concealed from the surrounding solvent. Consequently, σ_∞_ plateaus for surfactant concentrations above the CMC, since the bulk concentration of individual molecules remains close to the CMC.^25^ The CMC varies according to the chemical properties of each surfactant (e.g., its hydrophobicity) and solvent, in addition to physical properties like temperature. Surfactants are often described by the speed of their effect on the dynamic surface tension: a fast surfactant rapidly (∼1 ms) modifies the surface tension, while the opposite is true for a slow surfactant. Fast surfactants generally have high diffusion rates, but they must also be able to adsorb quickly once in the subsurface, overcoming an “adsorption barrier” arising from statistical and thermodynamic factors.^26^ In practice, the interface dynamics are assessed through the measurement of the dynamic surface tension.

Surfactants are used as spreading agents in inkjet printing, coating, and spraying because they improve droplet coverage by reducing the surface tension and thus increasing wettability.^29^ In these applications, during droplet impact, the existing free surface can be stretched, or brand new free surface can be formed, meaning that the surface excess concentration of surfactants becomes less than its equilibrium value—the dynamic surface tension characterizes the rate at which equilibrium is restored.^26^ It has been demonstrated that impacting surfactant-laden water droplets have a larger coverage than pure water, with the maximum spreading depending on the surfactant’s molecular weight, diffusion rate, and polarity.^30−37^ However, previous research, through both simulations and experiments, has indicated that the uneven distribution of surfactants on the droplet surface plays a crucial role in hindering spreading, primarily due to Marangoni stresses.^36,38^ Additionally, studies have revealed that Marangoni flows contribute to a delay in the entire spreading process.^39^ In contrast, if the surfactant distribution is uniform, surfactant-laden water droplets spread to a larger radius than pure water.^36^ In 2021, Hoffman et al. found that the dynamic surface tension plays a critical role in spreading and that the equilibrium surface tension is not relevant at impact timescales (a few milliseconds), concluding that only some fast-acting surfactants can influence droplet spreading.^33^ Regarding rough and superhydrophobic surfaces, Wang et al. found that surface roughness minimally affects the spreading diameter of dilute SDS droplets on micropillared arrays.^40^ However, higher surfactant concentrations decrease the droplet-receding velocity and can prevent bouncing on superhydrophobic surfaces.^37,41,42^ Additionally, surfactant molecules entering micro or nanostructures on superhydrophobic surfaces change the surface wettability, with faster impact speeds enhancing this interaction.^43−46^ Efforts at developing a prediction of maximum spreading ratios are numerous and take into account a variety of variables (fluid properties or wetting characteristics) but generally do not account for the unique properties of surfactant-laden liquids.^47−49^

Despite the widespread use of surfactants, their dynamics in situations where an impacting droplet splashes remain largely unexplored. The most relevant studies in this area have focused on the impact onto superhydrophobic leaves for agricultural applications. Vesicle surfactants have been shown to suppress *receding splashing* (where droplets breakup as the contact line recedes following maximum spreading) and bouncing on leaves by inducing a wetting transition during the inertial spreading stage to prevent receding (i.e., the droplet pins close to the maximum spread length), while the same work found that micelle surfactants do not have the same effect.^50^ Similar studies considering water droplet impact onto lotus leaves have found that spreading and receding splashing dynamics are somewhat correlated to the double-chain length of the surfactants involved.^34,51^ A related scenario is droplet impact onto pools, where small concentrations of surfactants can inhibit the formation, or pinch-off, of Worthington jets.^52^ However, in all of these works, the dynamics occur on timescales (≈2 ms and longer) that offer the fastest surfactants ample opportunity to adsorb at freshly formed free surfaces, unlike at the submillisecond timescales prevalent in “prompt” or “corona” splashing that are associated with the disintegration of the ejecta sheet immediately following impact.

In this work, we study the effect of various surfactants on the dynamics of impacting droplets *immediately* following impact, focusing on the fastest dynamics, including prompt splashing and early time spreading. In particular, using high-speed imaging and quantitative image analysis, we investigate the influence of the surfactant type (including ionic and nonionic) and concentration on the impact, spreading, and splashing of surfactant-laden water droplets on two substrates: Teflon and polystyrene. Surfactant solutions were characterized by measuring their dynamic surface tension on short timescales (including some submillisecond data). We have combined these measurements with the contact angle dynamics and the splashing ratio β to determine a parametrization that divides the splashing/no-splashing dynamics of surfactant-laden droplets and offers an insight into the effective surface tension on splashing timescales.

## Experimental Setup

In this work, we used four commercially available surfactants: sodium dodecyl sulfate (SDS), Triton X-100 (both purchased from Sigma-Aldrich), Surfynol 465 (free sample, Evonik), and BYK-3760 (free sample, BYK via Blagden Specialty Chemicals Ltd.). These surfactants were prepared in deionized water to produce six solutions, as detailed in Table 1. Their dynamic surface tensions were measured using a Sinterface BPA-2S maximum bubble-pressure (MBP) tensiometer (see below in this section), while their density and viscosity (at the concentrations used here) are similar to those of water (ρ = 997 kg m^–3^, μ = 0.93 mPa s).^33^ Temperature can have a significant effect on the surfactant properties (including their CMC), so all experiments were conducted at 23 ± 1 °C.

**Table 1:** Summary of the Surfactant Solutions Used in This Work

solution name	surfactant	type	molecular weight^27^	solution concentration
BYK	BYK-3760	polyether-modified polysiloxane	N/A	2.0 mass %
SDS 0.8 CMC	sodium dodecyl sulfate	anionic sodium salt	288.38 g mol^–1^	6.3 mM (0.8 × CMC)
SDS 1.3 CMC				10.5 mM (1.3 × CMC)
Surfynol	Surfynol 465	nonionic gemini surfactant	666 g mol^–1^	15.5 mM (1.3 × CMC)
Triton 1 CMC	Triton X-100	nonionic polyethylene glycol ether^28^	647 g mol^–1^	0.24 mM (1 × CMC)
Triton 20 CMC				4.8 mM (20 × CMC)

**Figure 1:**
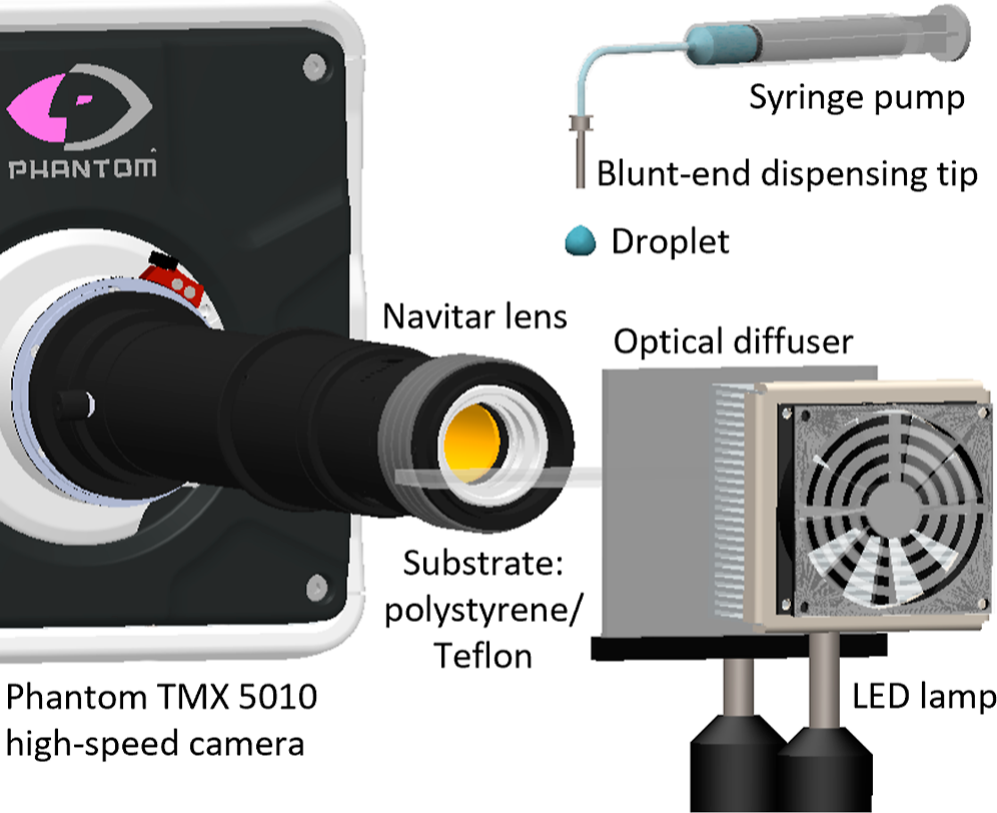
Schematic view of the experimental setup used to visualize the impact of droplets. Various systems were used in this work; the figure shows an example using a Phantom TMX 5010 high-speed camera equipped with a Navitar lens and lighting provided by a 100 W LED.

**Figure 2:**
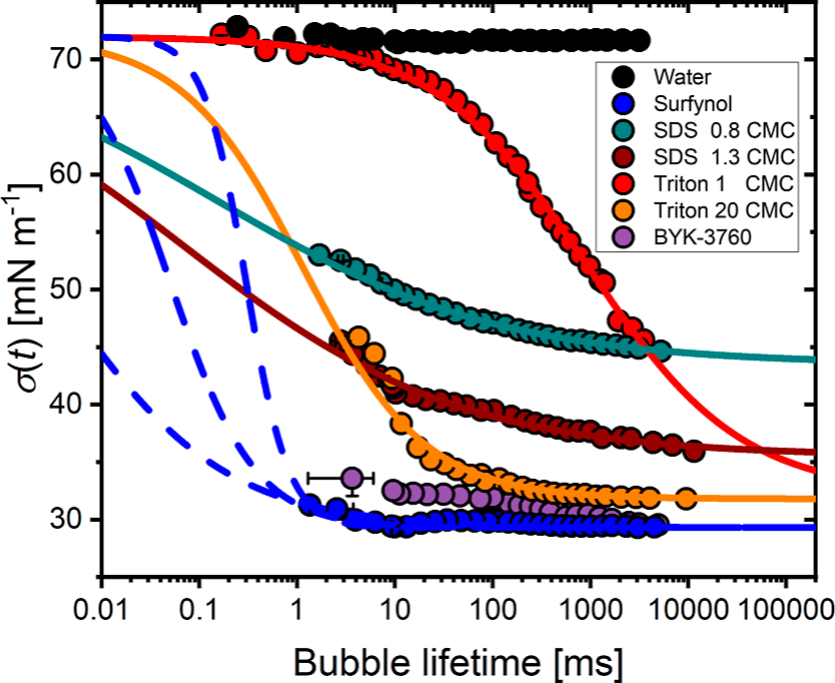
Dynamic surface tension for water and all surfactant solutions used in this work. Symbols indicate average data as measured by the maximum bubble-pressure tensiometer, for which error bars represent either 3 times the standard deviation or the largest difference observed by varying the critical point. Lines represent eq 2 applied to the data, either by a least-squares fit to the rapid fall region via eq 3 (solid lines) or manual fitting (dashed lines).

Our experimental setup is seen in Figure 1. Droplets were generated by dripping from stainless steel blunt-end dispensing tips, with outer diameters ranging from 0.31 to 1.27 mm (18–30 gauge, Metcal), resulting in drop diameters *D*_0_ from 1.9 to 2.9 mm. To ensure the surface tension of the impacting droplets was close to the equilibrium value for each surfactant solution, we established a minimum drop time formation of ≫15 s, before dripping. According to our MBP data (Figure 2, discussed below in this section), these dripping times are long enough that the surfactants can diffuse to, and adsorb at, a fresh free surface and reduce its dynamic surface tension from that of the solvent to be close to its equilibrium value (with the possible exception of Triton 1 CMC), as determined by pendant droplet tensiometery.^53^ After being dripped, the droplets impacted a dry flat solid polytetrafluoroethylene (PTFE/Teflon) or clear polystyrene substrate, which underwent multiple rounds of rinsing and drying prior to each experiment to ensure the substrate was surfactant-free on impact. The dispensing tip height varied to adjust *U*_0_ from 0.87 to 4.88 m s^–1^, which was measured using an in-house MATLAB script based on a second-order polynomial fit to the droplet position. *D*_0_ is determined based on the radius of curvature at the south pole immediately before impact, as explained in our previous publication,^20^ which accounts for any nonsphericity of the droplet induced by gravity or capillary waves.

**Figure 3:**
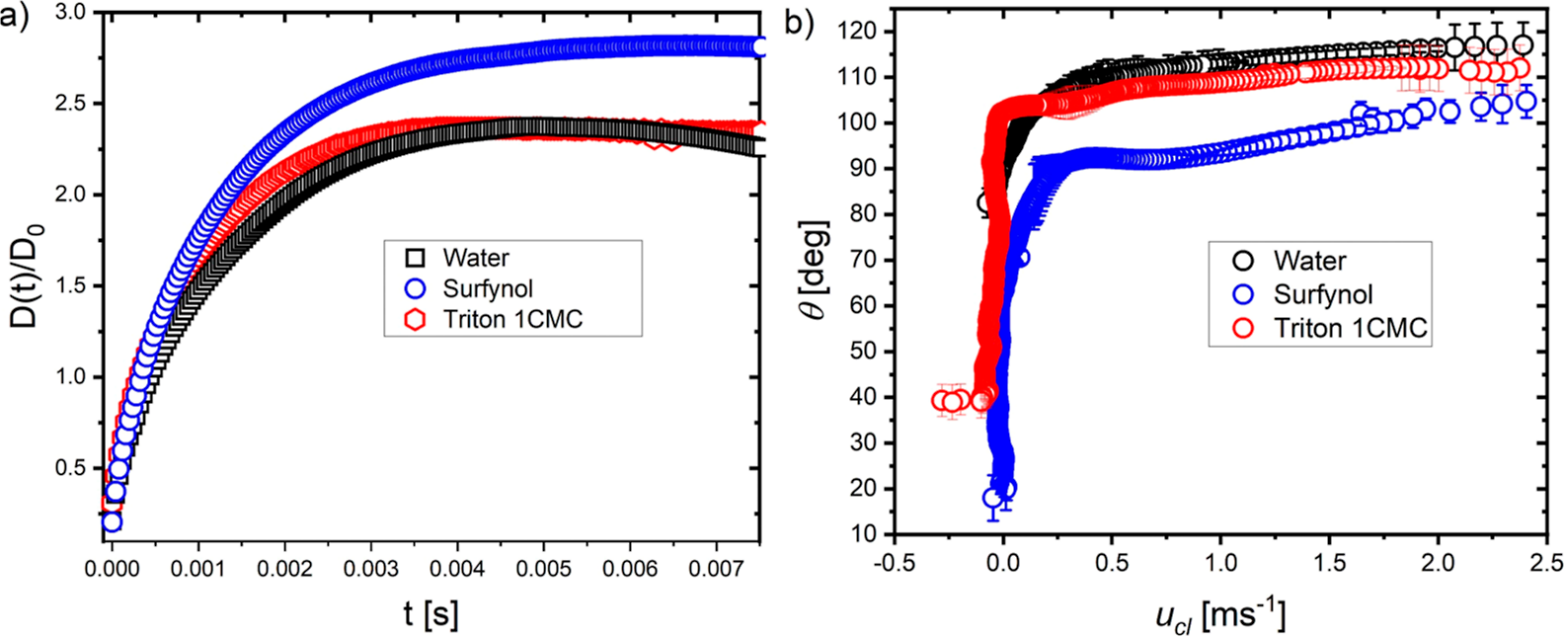
Spreading dynamics following the droplet impact on *polystyrene*. (a) Evolution of the spreading diameter *D*(*t*)/*D*_0_ in terms of the time from impact. As observed, the spreading diameter of the Triton 1 CMC (a slow surfactant) is similar to that of water. In contrast, Surfynol (a fast surfactant) presents larger maximum spreading and equilibrium diameters. (b) Dynamic contact angle θ_*D*_ in terms of the contact line velocity (*u*_cl_). As seen, at *u*_cl_ > 2 m s^–1^, the contact angle is not the same for all the solutions, i.e., θ_*D*_ = 108 ± 8° for Triton. The impact velocity for these experiments is *U*_0_ = (0.88 ± 0.02) m s^–1^.

Droplet impacts were imaged with a Phantom v710, a v2512, or a TMX-5010 high-speed camera in a shadowgraphy configuration. The cameras were equipped with either a Navitar 12× zoom lens (with a 2× F-mount adapter) or a Laowa 5× Ultra-Macro 25 mm lens. Recording speeds ranged from 23,000 to 78,000 frames per second (fps). Droplets were backlit by a 100 W LED source or a 89 North Photofluor II lamp, enabling exposure times in the range of 0.2–5.0 μs. Under these configurations, the effective resolution ranged from 81 to 250 pixels mm^–1^, the highest resolution being used to measure the dynamic contact angle. These images were analyzed in-line with our previous work.^54^ In brief, for the contact angle analysis, an image is first binarized to detect the droplet boundary. The contact point is identified as the first black pixel in an otherwise white background at a line where the substrate would be located. This line is found by finding the cusp made by the image of the drop and its reflection on the substrate. A second-order polynomial is fit to a fraction of the droplet boundary near the contact line with the least-squares method, the boundary normally representing 4% of the overall perimeter. The tangent to the polynomial is then evaluated at the contact point to obtain the contact angle. The algorithm takes all the images from a high-speed sequence to obtain the dynamic contact angle; an example of these results are seen in Figure 3.

## Results and Discussion

### Dynamic Surface Tension

Dynamic surface tension measurements were obtained using an MBP tensiometer (Sinterface BPA-2S) in a surface lifetime range of 0.2 × 10^–3^–11.5 s. The lifetime of the bubble in MBP tensiometry approximates the age of its free surface, from a presumed starting point with no adsorbed surfactant. Under the standard MBP method, air bubbles are produced at the tip of a capillary within the liquid sample at constant flow rates. The gas pressure is monitored to identify its maximum value, which coincides with the time when the bubble has a hemispherical shape. Surface tension is then calculated from the measured maximum pressure using the Young–Laplace equation, with corrections applied for gravitational effects, capillary and aerodynamic resistance, and viscosity. We refer the reader to Section 5 of Fainerman and Miller^55^ (in particular, eqs 1–5) for a full description of the underlying equations and iterative methods used in the standard MBP tensiometry method implemented in the BPA-2S. Most commercially available tensiometers only operate using this standard MBP method, so only report surface tension applicable to surface ages longer than approximately 10 ms, for which a direct measurement of the bubble lifetime is feasible. This measurement is typically made either from the oscillations of the measured pressure or (as in the BPA-2S) oscillations of the gas flow fed to the capillary; the latter has been shown to be more reliable.^56^

Evaluating the dynamic surface tension at shorter lifetimes than 10 ms is critical for understanding impacting droplets, given that splashing happens well within the first 2 ms of impact.^14^ Few techniques are available to measure the dynamic surface tension on such timescales, though it was recently suggested that data at millisecond timescales can be inferred from the dynamics of the droplet impact on hydrophobic surfaces.^33^ Dynamic surface tension measurements with bubble lifetimes as short as 0.1 ms are however available with the tensiometer used in this work via an extension to the standard MBP method based upon the transition between the formation of bubbles (at low gas flow rates) and a gas jet (high flow rates leading to lifetimes ≪10 ms). Briefly, the bubble deadtime (the period between the bubble maximum pressure and the formation of the subsequent bubble) required for the formation of bubbles (as opposed to a gas jet) can be determined analytically from Poiseuille’s law. The resulting equation can be reduced to a simpler form containing only experimentally attainable parameters (contrast eqs 15 and 19 in ref (55); eqs 67 and 68 in ref (57)) by keeping the bubble volume constant using a deflector placed at a known fixed distance from the capillary tip.^57^ Hence, bubble lifetimes can be determined indirectly, thereby overcoming the high flow rate limitations of direct measurement. For this method, the flow rate and pressure at the “critical point” of the transition between the bubbles and gas jet regimes are required, which can be determined algorithmically from the pressure versus gas flow rate data,^58^ along with the bubble deadtime. The latter depends on geometric parameters related to the capillary and so can be considered constant (though it is directly measured in BPA-2S for lifetimes greater than 10 ms). The use of a deflector also shortens the deadtime to around 10 ms, which is required for submillisecond measurements.^59,60^ Indeed, the limitation of 0.1 ms in this extended MBP method arises from the deadtime and gas flow rate precision.^59^ The extended MBP method is also accurate only at very short lifetimes for low-concentration solutions, so measurements down to 0.1 ms are therefore not necessarily achievable for all surfactant solutions. Reliable submillisecond data can usually be accurately obtained for solutions with a dynamic surface tension at lifetimes of  comparable to that of the solvent (e.g., Triton 1 CMC, see Figure 2) at most solution concentrations. The dynamics of the bubbles at high flow rates (giving rise to a shorter effective deadtime than the physical one) can also affect the suitability of this method at low lifetimes, especially for concentrated solutions (e.g., Triton 20 CMC, for which reliable data could be obtained for lifetimes >1 ms).^60^

Figure 2 presents the dynamic surface tension measurements obtained from the standard and extended MBP methods for water and all surfactant solutions used in this work (see Table 1). We supplement these MBP data by the empirical formula introduced by Hua and Rosen^61^
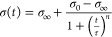
2where σ_∞_ is the equilibrium surface tensions of the solution, σ_0_ is the surface tension of the solvent (water, σ_0_ = 72.4 mN m^–1^), *t* is the surface age (bubble lifetime), τ is the characteristic time taken by the surfactant molecules to reach the surface of the liquid, and *n* is a fitting parameter; eq 2 describes dynamic surface tension curves (lines in Figure 2), as measured by MBP tensiometry. Several authors have suggested that this formula can be used to extrapolate MBP data to shorter lifetimes relevant to droplet impact dynamics.^33,62^ In practice, τ approximates the surface age *t*_1/2_ at which the surface pressure is 0.5 (σ_0_ – σ_∞_), and *n* alters the curve slope; eq 2 can be expressed in a logarithmic form as
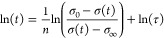
3which represents a linear equation where ln(τ) and 1/*n* can be calculated from empirical data as fitting parameters. However, as noted by Hua and Rosen,^61^ this fitting results in large errors when the ratio (σ_0_ – σ)/(σ – σ_∞_) is close to 0 (when σ = σ_0_) or ∞ (when σ → σ_∞_). Therefore, we restrict our fitting of eq 2 (via eq 3) to MBP data lying in the “rapid fall region” defined by 0.1 < (σ_0_ – σ)/(σ – σ_∞_) < 10, which produce the solid lines in Figure 2 and may be used to predict the surface tension at shorter lifetimes.

The results shown in Figure 2 show key differences between the surfactant solutions. For example, Triton 1 CMC may be regarded as a slow surfactant,^33^ as a long time is required to reach its equilibrium surface tension value, and its dynamic surface tension is approximately that of water for surface ages less than 5 ms. The fitting of eq 2 is therefore very reliable, as there is little ambiguity in *t*_1/2_ (which sets τ) and the slope there (which sets *n*). Confirming this notion, the data (σ_0_ – σ)/(σ – σ_∞_) < 0.1 (which were not part of the fitting) are seen to be in good agreement with the fitted model. For the high-concentration Triton and both SDS solutions, while the MBP data do not reach surface tensions close to σ_0_, there are a sufficient number of data points in the rapid fall region to fit eq 2 and hence predict the surface tension at shorter timescales with confidence. These predictions will be explored to analyze droplet impact and splashing dynamics in the following sections. It is however notable that most data points in the rapid fall region of Triton 20 CMC lie in the time domain inaccessible to most MBP tensiometers (less than 10 ms); without these points, the fitting of eq 2 for Triton 20 CMC (or any faster surfactant solution with the rapid fall region within the microsecond timescale) would be unreliable.

In contrast, BYK-3760 and Surfynol are fast surfactant solutions, attaining a dynamic surface tension close to the equilibrium value at a surface age of a few milliseconds. As seen in Figure 2, for these surfactant solutions, we therefore have no MBP data in the rapid fall region, leading to ambiguity in *t*_1/2_. Hence, τ cannot be determined unequivocally. Moreover, a wide range of τ and *n* values closely approximate the available MBP data for such surfactant solutions, as demonstrated by three manual fits of eq 2 (τ = 3 × 10^–1^ ms, *n* = 2; τ = 5 × 10^–2^ ms, *n* = 1.0, τ = 3 × 10^–3^ ms, *n* = 0.5) represented by dashed lines in Figure 2. While these three fits are consistent with the available MBP data, they lead to very different predictions of the dynamic surface tension at lifetimes less than 1 ms. We also attempted least-squares fitting to the Surfynol data for lifetimes outside the rapid fall region but found that the fitted values of τ and *n* were very sensitive to the number of data points included (see Figure S1, Supporting Information).

As warned by Hua and Rosen, and confirmed by our results, the surfactant behavior affects the applicability of eq 2 to reliably predict the surface tension at short timescales. In particular, we conclude that, in agreement with Hua and Rosen, the ability of eq 2 to reliably predict the surface tension at short timescales depends on having sufficient MBP data in the rapid fall region. In the context of this work, the method is able to reliably predict the surface tension of the Triton and SDS solutions in the microsecond lifetime range but not Surfynol and BYK-3760. Notably, it is only the extension of the MBP method to lifetimes less than approximately 10 ms (inaccessible with the standard MBP method) that provides us with sufficient data in the rapid fall region to make a reliable submillisecond prediction for Triton 20 CMC.

### Droplet Impact

**Figure 4:**
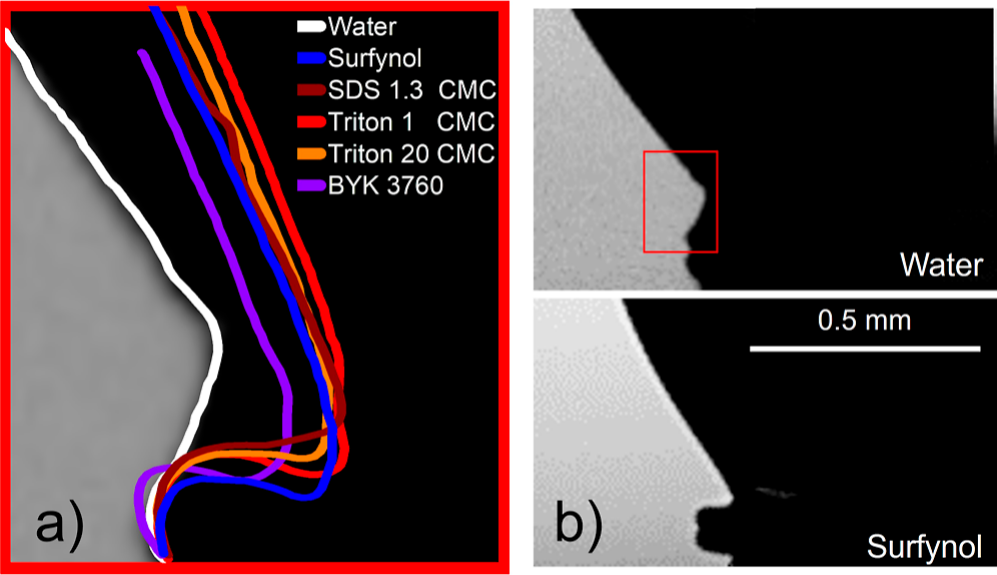
Droplet free surface near the contact line, 0.2 ms after impact. (a) Profiles after impact on Teflon at *U*_0_ = (1.20 ± 0.06) m s^–1^, overlaid onto a photo of water, where the contact line speed is *u*_cl_ = (5.54 ± 0.25) m s^–1^. (b) Photos in the vicinity of the contact line for water (top) and Surfynol (bottom).

We performed droplet impact experiments over a large range of impact speeds to obtain conditions from smooth spreading to splashing. Following past conventions,^14^ we performed impact experiments at *U*_0_ = 0.8 to 1.2 m s^–1^ to determine the dynamic contact angle of all the surfactant-laden solutions on our two substrates: polystyrene and Teflon. At this impacting speed, only smooth spreading conditions (i.e., no breakup) are found for all solutions. We note that at times *t* < 0.5 ms, the measurement of dynamic contact angles are inaccurate given the effective resolution of our images. Therefore, to investigate the spreading of surfactant-laden droplets at these short timescales, we look at individual droplet profiles to provide a qualitative comparison. These profiles at *t* = 0.2 ms are shown in Figure 4. As seen, for all surfactant-laden droplets, the lamella is created faster than for pure water. Despite the faster lamella formation, the contact diameter *D*(*t*)/*D*_0_ remains equal, within experimental error, and the droplet free surface shape is different at this short timescale. This observation may be unexpected a priori as, at this submillisecond timescale, most of the surfactant solutions studied here have a dynamic surface tension similar to the solvent (see Figure 2). This is especially true for the slow surfactant solutions (e.g., Triton 1 CMC), as identified in the Experimental Setup section.

We argue that, at this speed and timescale, there is no new surface area creation, i.e., the droplet surface area is deformed without creating a significant new surface. Thus, smooth spreading occurs in a liquid interface that has the equilibrium surface tension of the droplet. The effects of surfactants on the droplet profile can also be observed at *t* = 0.5 ms, where a capillary wave develops in the water droplet but not for Surfynol (see Figure S2 in the Supporting Information). In fact, this capillary wave is suppressed for all the surfactant solutions. We theorize that the capillary wave is suppressed by the reduction in surface tension and surface rigidification.^23^

### Spreading Diameter and Dynamic Contact Angle

We now contrast the spreading diameter *D*(*t*) of the surfactant solutions against that of water. Figure 3a summarizes our results where we show the spreading diameter of water, Triton 1 CMC (slow surfactant), and Surfynol (fast surfactant). The data for all the other solutions can be found in Figure S4 in the Supporting Information. As seen, there is little variation at short timescales; the spreading only differentiates after 1.0 ms. In fact, Triton 1 CMC and water have similar spreading diameter dynamics where the kinematic, spreading, relaxation, and equilibrium phases are readily observable (Figure 3a). The effect of the surfactant can be mostly observed at later stages, where receding is suppressed, and the equilibrium diameter remains larger than that for water. Of all the liquids, Surfynol has the largest maximum spreading diameter (Figure 3a), where receding or surface resistance has been suppressed. This observation is consistent with the dynamic surface tension measurements, as Surfynol is the surfactant that has the lowest value across all surface ages. This is in line with previous results that showed that *fast* surfactants alter the maximum spreading diameter, while *slow* surfactants do not have the time to modify the spreading dynamics.^33^

The dynamic contact angles for water, Triton 1 CMC, and Surfynol on polystyrene in terms of the contact line velocity (*u*_cl_) are shown in Figure 3b. The dynamic contact angles for all of the liquids on polystyrene and Teflon are shown in Figures S4b and S5b in the Supporting Information. Figure 3b shows that the dynamic contact angles for Triton 1 CMC and water are in agreement with each other within error bars. In contrast, for Surfynol, θ_*D*_ is lower at all times, as the surface tension of Surfynol is smaller than that for Triton 1 CMC and water even at the first instants of spreading of spreading. Moreover, at *u*_cl_ = 0.25 m s^–1^, the difference in θ_*D*_ between Surfynol and water is ≈15°. This is expected, as according to our tensiometer measurements, Surfynol has the lowest surface tension at the moment of maximum spreading (*t* ≈ 1.0 ms). Concluding, Surfynol has the smallest θ_*eq*_, while water has the highest. In general, θ_*eq*_ depends on the nature of the surfactant and its concentration.

### Splashing Dynamics

Here, we report our findings for high impact velocities (*U*_0_ = 2.0–4.8 m s^–1^), which typically lead to splashing for droplets impacting moderately hydrophobic substrates like Teflon and polystyrene. Crucially, we recorded at up to 78,000 fps to reveal the initial splashing dynamics that might be missed at lower frame rates, which also confirmed that the onset of splashing occurs well within the first 0.1 ms following impact. Notably, this timescale is shorter than the minimum surface age achievable with commercially available tensiometers, which limits our ability to independently assess the dynamic surface tension of a free surface with this age.

**Figure 5:**
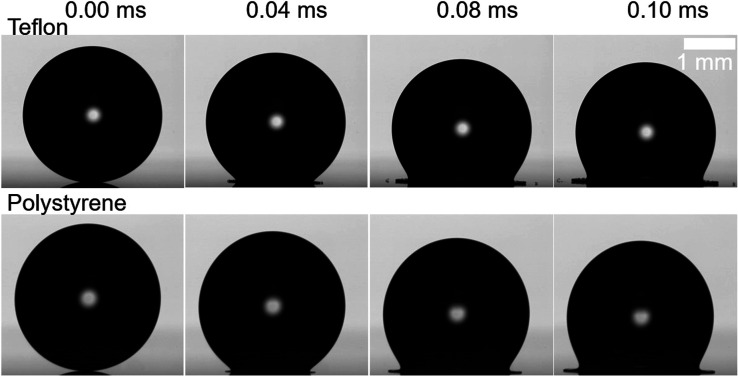
Near-identical Triton 1 CMC droplets impacting Teflon (top) and polystyrene (bottom) at *U*_0_ = (3.07 ± 0.02) m s^–1^. Splashing is observed for the impact on Teflon, while no secondary droplets emerge after the impact on polystyrene. This result indicates that substrate wettability via the dynamic contact angle influences the splashing behavior of surfactant-laden droplets, similar to simple fluids.

**Figure 6:**
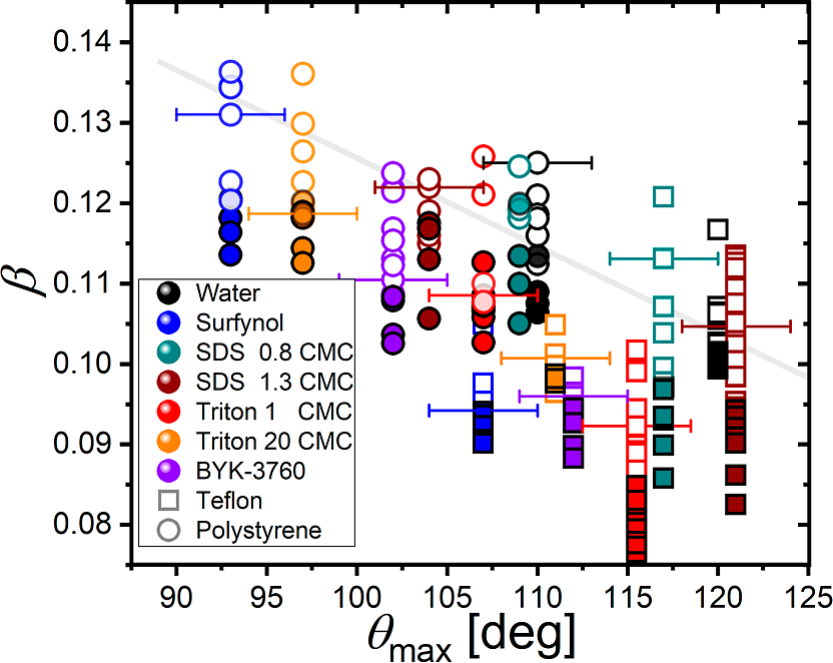
Splashing for droplets containing surfactants impacting polystyrene and Teflon. The plot shows the splashing parameter β in terms of θ_max_. Here, splashing is denoted by open symbols, while closed symbols represent no splashing. Representative error bars are shown for some data points for simplicity; vertical error bars are much smaller than the size of the symbol.

Past studies on surfactant-free Newtonian liquids have successfully parametrized droplet splashing by using the splashing ratio β (as defined in eq 1 of the Introduction section). In addition, it has also been established for such simple fluids that wettability plays a role in splashing via the dynamic contact angle: the higher the dynamic contact angle, the lower the critical β required to transition from no splashing to splashing.^14^ To confirm whether wettability also affects the splashing threshold of surfactant solutions, Figure 5 shows the impact of near-identical Triton 1 CMC droplets on two different substrates: the droplet splashes on Teflon but smoothly spreads, without splashing, on polystyrene. In particular, the splashing threshold for simple fluids is given by
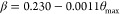
4which is the solid gray line in Figure 6. The behavioral difference seen in Figure 5 is consistent with their different dynamic contact angles (θ_max_ = 116° for Teflon and θ_max_ = 108° for polystyrene), which indicates that eq 4 can also be used to understand surfactant-laden droplet splashing.

As previously mentioned, β depends on the surface tension, which for simple fluids is constant (i.e., the equilibrium surface tension). In the case of surfactant-laden droplets, one expects β to be evaluated with the effective surface tension value of the interface near the lamella (where splashing originates) at the onset of splashing. Considering that a portion of the lamella originates as a thin sheet ejected from the surrounding fluid near the point of contact between the droplet and the substrate, it is uncertain a priori whether the lamella consists of a *freshly formed* (new) free surface initially lacking surfactant or if it is formed of a *stretched* free surface (of the impacting droplet) that retains some, or all, of the original surfactant distribution. In the former case, any surfactant affecting splashing would need to be adsorbed between the lamella ejection and the onset of splashing (i.e., within 0.1 ms). In the latter case, the surfactants are already adsorbed at the surface, though their concentration may be being diluted due to the creation of a new surface. In the former case, we would not expect a significant effect of the surfactants on the splashing threshold, especially for slow surfactant solutions (such as Triton 1 CMC) that have a dynamic surface tension at the splashing timescale close to that of water. However, in the latter, a difference in the splashing behavior between surfactant solutions and its solvent is expected.

**Figure 7:**
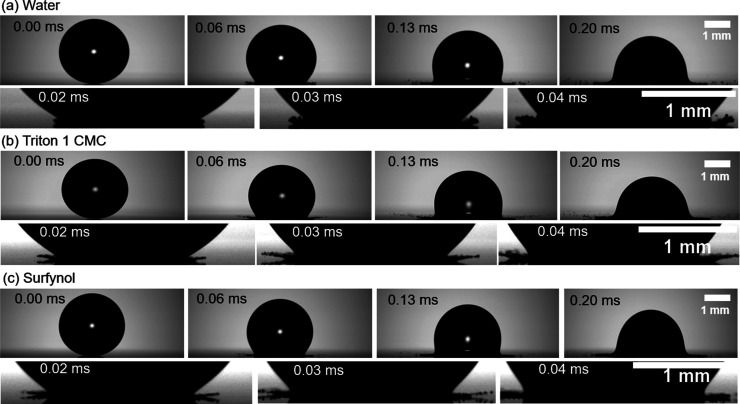
Impact and splashing of surfactant-free and surfactant-laden droplets onto Teflon, with a constant splashing ratio of β = 0.109 ± 0.002. Snapshot sequence capturing the initial 0.05 ms of splashing dynamics for (a) water, (b) Triton 1 CMC, and (c) Surfynol. The images in the first row captures the entire sequence of events from the moment a droplet impacts a surface, showing the splashing behavior until 0.20 ms, which fully illustrates the splashing phenomenon. Meanwhile, the second row of images depicts the initial stages of splashing, including the moment when the first fragment detaches from the main droplet, highlighting the behavior of the forming lamella.

Looking at the splashing dynamics from our ultrahigh speed images in Figure 7, we see that the ejection of the lamella from surfactant-laden droplets (Triton 1 CMC and Surfynol here) exhibits notable differences compared to that of water, with the initial lamella appearing thicker and ejecting less satellite droplets for water. These differences are also reflected in the critical splashing ratio. In Figure 6, to facilitate comparison between different surfactants, we have used the fixed surface tension value of the solvent (water, σ = 72.4 mN m^–1^) to evaluate β in all of the cases. Since water is the solvent of all fluids reported in this work, its surface tension value should divide the impact outcomes according to eq 4 if surfactants had no effect on the surface tension on splashing timescales. However, Figure 6 shows that the critical β for splashing is *lower* than the value predicted by eq 4 for all surfactants and both substrates. Hence, these surfactant-laden droplets splash at a lower impact speed than that of water. Most notably, the splashing behavior of Triton 1 CMC and water is different, despite having the same dynamic surface tension for surface ages comparable with the onset of splashing. This observation strongly indicates that the lamella (at least partially) contains adsorbed surfactants, so it is likely a stretched version of the original free surface in equilibrium. In our experiments, droplets were generated by dripping slowly enough that the droplets reached their equilibrium surface tension while pendant and therefore can be assumed to have a surface with surfactants in equilibrium just prior the impact. Given the difference in behavior, we can conclude that the surfactant adsorbed *prior* to impact affects splashing postimpact.

**Figure 8:**
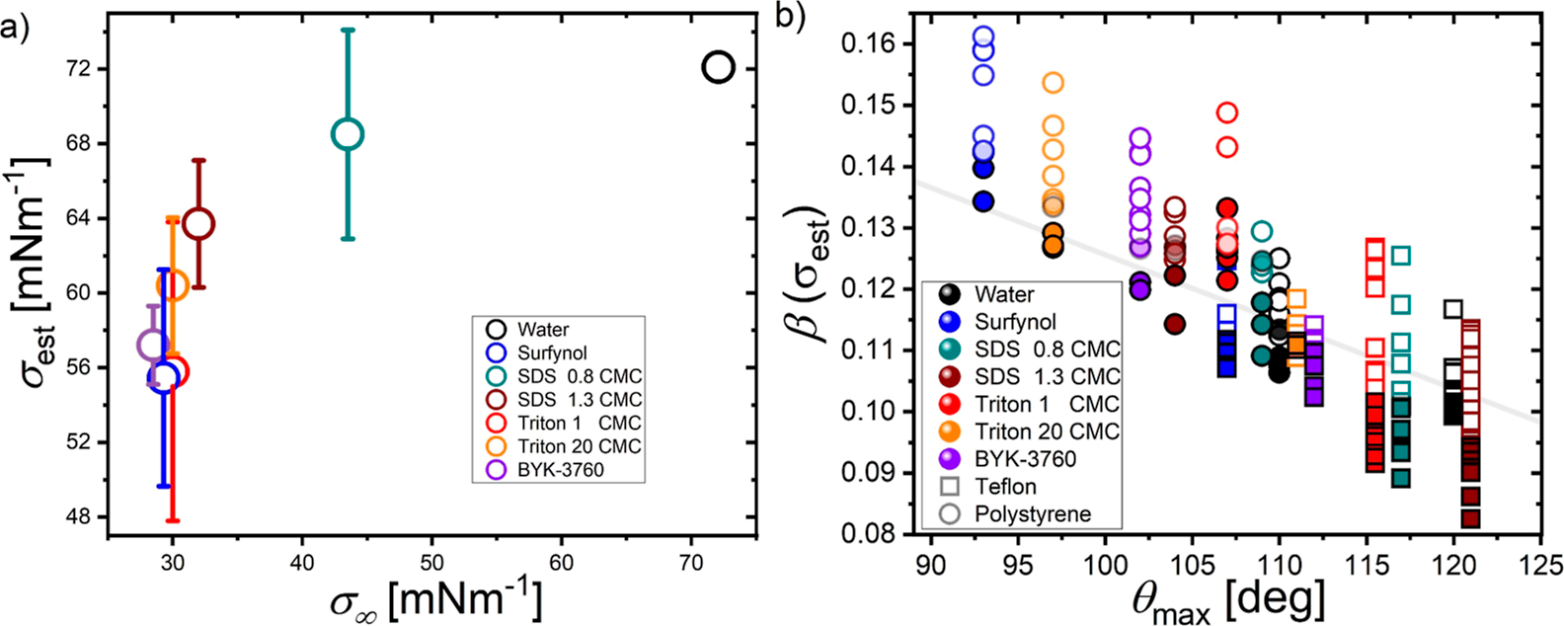
(a) Predicted prevailing surface tension σ_est_ at the onset of splashing in terms of the equilibrium surface tension σ_0_. The prevailing value σ_est_ is calculated such that the splashing threshold of each surfactant matches that of eq 4. In all cases σ_∞_ < σ_est_ < σ_0_. (b) Reconstruction of Figure 6: The β value has been calculated by employing σ_est_ (surface tension at the onset of splashing) in eq 1.

For Surfynol, substituting its equilibrium surface tension (σ_∞_ ≈ 30.0 mN m^–1^, as opposed to that of water) into β does not recover the splashing threshold line given by eq 4 doing so would yield a critical β around 80% higher compared to that shown in Figure 6. In contrast, assuming a surface tension similar to that of water, the critical β indicates a surfactant-induced reduction in splashing propensity of around 15%, corresponding to a significant reduction in the critical impact velocity (around 20%). Since β ∝ σ^–2/3^, this observation indicates that surfactants lower the prevailing surface tension at the onset of splashing. This is consistent across all of our surfactant solutions. In other words, slower impact velocities are required for splash surfactant solutions; this lower threshold arises from dynamic surface tension differences between the solutions and the solvent. Differences on the splashing threshold can be used to estimate the surface tension prevailing at the onset of splashing, σ_est_, by quantifying the vertical shift required to match eq 4. The result of this exercise is seen in Figure 8a, where we plot the predicted prevailing surface tension against the equilibrium value for each solution, and in Figure 8b the original data but replotted using this σ_est_ value in β. As seen, for a given solution, σ_est_ generally increases with σ_∞_. We suggest that σ_est_ stems from the surfactant dilution (already equilibrated) at the free surface during the stretching of the droplet from spherical on impact; this is in agreement with σ_est_ being in between the equilibrium and solvent surface tensions, i.e., σ_∞_ < σ_est_ < σ_0_. This mechanism does not rely on adsorption or desorption of surfactant on short timescales, so we would not expect the precise surfactant type (chain length, polarity, etc.) or aggregate structure (vesicle or micellar) to have a significant effect, such as that has been found in previous works for longer timescale dynamics like receding splashing. This inference is consistent with our experiments as Surfynol and Triton 1 CMC splash with similar β values (using σ = σ_0_) because they have a similar equilibrium surface tension σ_∞_, despite having different molecular structures and short timescale dynamic surface tensions. We note that this proposed mechanism does not preclude the possibility of additional surfactants being adsorbed and desorbed at the free surface during impact. However, it does explain how all surfactants that reduce the equilibrium surface tension of a fluid significantly can influence very short timescale splashing behavior, including those solutions (e.g., Triton 1 CMC) for which the dynamic surface tension on such timescales is the same as that of the solvent.

## Conclusions

Our experiments constitute a thorough exploration of the dynamics of droplet splashing in the presence of surfactants. Notably, the splashing behavior shows significant distinctions between the surfactant-laden droplets and pure water. We observed that, upon the impact of a surfactant-laden droplet at its equilibrium surface tension, the resulting splashing behavior is related to the equilibrium surface tension of the solution due to stretching of the droplet to form a lamella. This effectively dilutes the free surface concentration of the adsorbed surfactant when the droplet is stretched to form a lamella on impact. A similar effect has been recently reported in experiments simulating the breaking dynamics of ocean waves (plunging breakers), in the context of ocean waves where surfactants may play a crucial role.^63^ Therefore, splashing behavior is primarily determined by surfactants that are already adsorbed on impact, rather than surfactants adsorbed postimpact. The specific value is determined by the equilibrium surface tension, implying that surfactant solutions with a lower equilibrium surface tension tend to splash more easily, assuming that the free surface of the impacting droplet is equilibrated. The behavior holds true regardless of the dynamic surface tension at typical submillisecond splashing timescales, the type of the surfactant, or aggregate structure. Furthermore, our investigations have highlighted notable distinctions between surfactant solutions and the solvent with regard to spreading diameter, lamella ejection, and splashing. Specifically, surfactants influence the shape adopted by surfactant-laden droplets during the process of lamella formation. A significant revelation from our study is the distinct difference in spreading behavior between droplets containing fast and slow surfactants, with the former exhibiting a wider coverage area upon impact. These findings significantly enhance our understanding of the effects of surfactants on droplet impact, with broad implications across diverse applications where these additives are present, whether intentionally or unintentionally, in industries such as inkjet technology, spraying, and agriculture.

## Supporting Information Available

Fitting parameters (*n* and τ) for Figure 2; least-squares fitting of the dynamic surface tension of Surfynol; zoomed-in images and descriptions of the postimpact capillary wave suppression; measurements of the dynamic contact angle as a function of time for various mixtures; plots of the spreading diameter and the dynamic contact angle, in terms of time and the speed of the contact line; and measurements of the maximum spreading factor against other existing models PDF
