# Supplementary material for: Effect of Surfactants on the Splashing Dynamics of Drops Impacting Smooth Substrates
Source: Langmuir. 2024 Mar 6;40(17):8781–90. doi: 10.1021/acs.langmuir.3c03248 (PMC11064227; doi:10.1021/acs.langmuir.3c03248)
Supplement: Supplementary file 1 — la3c03248_si_001.pdf [file la3c03248_si_001.pdf]

Supporting Information: The effect of surfactants on the splashing dynamics of drops impacting smooth substrates

Nonu Varghese,[1,2] Thomas C. Sykes,[3] Miguel A. Quetzeri-Santiago,[3,4] Alfonso A. Castrejón-Pita,[3] and J. Rafael Castrejón-Pita*[2]

[1)] *School of Engineering and Material Sciences, Queen Mary University of London, E1 4NS, United Kingdom.*

[2)] *Department of Mechanical Engineering, University College London, Torrington Place, London, WC1E 7JE, United Kingdom*

[3)] *Department of Engineering Science, University of Oxford, Oxford, OX1 3PJ, United Kingdom*

[4)] *Instituto de Investigaciones en Materiales, Universidad Nacional Autónoma de México, Cd. Universitaria, Mexico City, 04530, Mexico*

(*Electronic mail: r.pita@ucl.ac.uk)

Number of pages: 5

Number of figures: 6

Number of tables: 1

EMPIRICAL DYNAMIC SURFACE TENSION MODEL

Fitting parameters used in Figure 2 (of the main text)

As seen in Figure 2 of the main text, the model of Hua and Rosen (Equation 2) was applied to our dynamic surface tension data, either by a least squares fit to the rapid fall region (defined by $0.1 < (\sigma_0 - \sigma)/(\sigma - \sigma_\infty) < 10$) to determine τ and n via equation 3, or manual fitting (dashed lines). The solvent surface tension $\sigma_0 = 72.4\,\mathrm{mN\,m^{-1}}$ (that of water) and equilibrium surface tension σ_∞ are parameters that need to be measured. In this work, σ_∞ was determined using the bubble pressure tensiometer (those surfactants for which the gradient at large surface ages is zero, such as Surfynol) or pendant droplet tensiometry. The fitted values of τ and n (alongside σ_∞) underpinning Figure 2 are given in Table S1.

TABLE S1. Fitting parameters n and τ, alongside the corresponding equilibrium surface tension σ_∞, underpinning the model fits seen in Figure 2 (main text). Given that the surfactant were aqueous, a fixed solvent surface tension of $\sigma_0 = 72.4\,\mathrm{mN\,m^{-1}}$ was used.

Solution name	σ_∞ ($\mathrm{mN\,m^{-1}}$)	τ (ms)	n
SDS 0.8 CMC	43.5	0.15	0.30
SDS 1.3 CMC	35.5	0.07	0.31
Triton 1 CMC	32.3	914.83	0.55
Triton 20 CMC	31.8	1.15	0.70
		3×10^{-3}	0.50
Surfynol	29.3	5×10^{-2}	1.00
		3×10^{-1}	2.00

Least squares fit to the Surfynol MBP data

Here, we further explore the ability of the Hua and Rosen model (Equation 2 in the main text) to accurately predict $\sigma(t)$ at shorter bubble lifetimes (usually interpreted as a measure of surface age) than the available MBP data. For Surfynol, the equilibrium surface tension is $\sigma_\infty = 29.3\,\mathrm{mN\,m^{-1}}$, whilst the surface tension of the water is $\sigma_0 = 72.4\,\mathrm{mN\,m^{-1}}$, so the rapid fall region (defined in the main text) for Surfynol is $[33.22, 68.48]$ $\mathrm{mN\,m^{-1}}$. However, the measured data for bubble lifetimes between $1.0\,\mathrm{ms}$ and $4.5\,\mathrm{s}$ is $\sigma(t) \in [29.3, 31.2]$ $\mathrm{mN\,m^{-1}}$, so the available MBP data for Surfynol does not include data *any* in the rapid fall region. We therefore could not fit the model using the

procedure described in the main text (solid lines in figure 2).

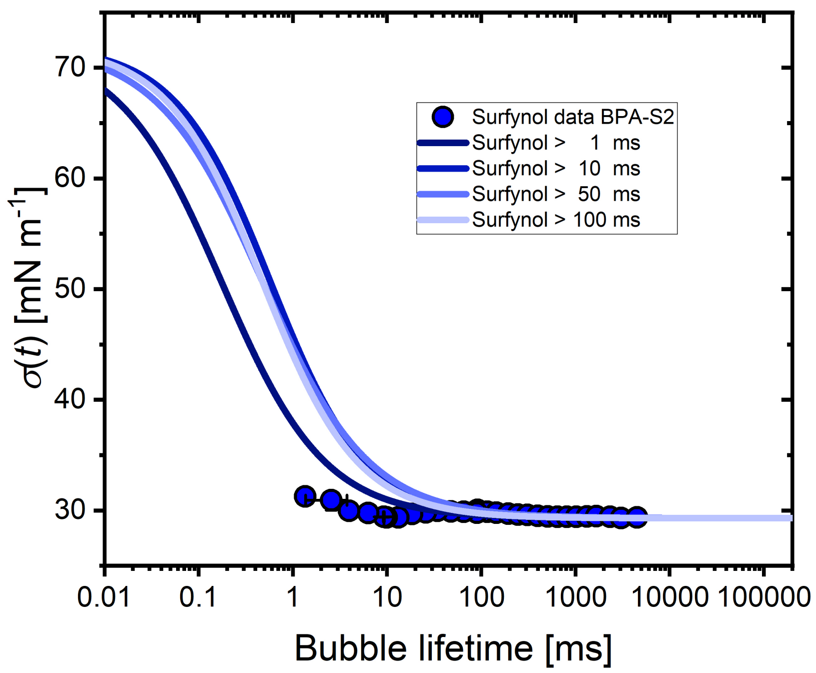

FIG. S1. Least squares fit of equation 2 (main text) to the Surfynol dynamic surface tension data obtained with the BPA-2S with equation 2 in the main text. Different ranges of experimental points were used to fit equation 2, as indicated in the legend (e.g. for 'Surfynol > 50 ms', only points representing a surface age of greater than 50 ms were fit).

Instead, here we found τ and n by least-squares fitting to *all* measured MBP data (dark blue line in Figure S1). To determine the robustness of this fit, we made several other least-squares fits excluding different data points. As seen in Figure S1, we also fit only the data with surface ages larger than 10 ms (this is the data that would be available using only the standard MBP method – i.e. the output of most commerical bubble pressure tensiometers), 50 ms and 100 ms, finding that the fitted values of τ and n are sensitive to the number of data points included. In particular, the availability of shorter lifetime data 1 ms − 10 ms in our work thanks to the extended MBP method (described in the main text) shifts the predicted rapid fall to the left in Figure S1 (by reducing τ), demonstrating that the predicted dynamic surface tension depends critically depends on the lowest surface age available when the fit is based on data outside the rapid fall region. This dependence results from there being no information encoded in the data to indicate where the rapid fall should be (i.e. the value of τ). We must therefore conclude that predictions of $\sigma(t)$ at shorter bubble lifetimes than measured are not reliable when they are based on least squares fitting of MBP data solely outside the rapid fall region.

SPREADING AND CONTACT ANGLE DYNAMICS

Capillary Wave

Figure S2 shows the profile of the droplet lamella at $t = 0.5$ ms. At this time a capillary wave develops in water, while is suppressed for all the surfactant solutions. We argue that this capillary wave is suppressed by the reduction in surface tension and surface rigidification caused by the surfactants.

Dynamic contact angle

Figure S3 illustrates the dynamic evolution of the contact angle over time, following the impact of a drop onto polystyrene. The provided data comprises raw measurements, with the contact angle values linked to the respective

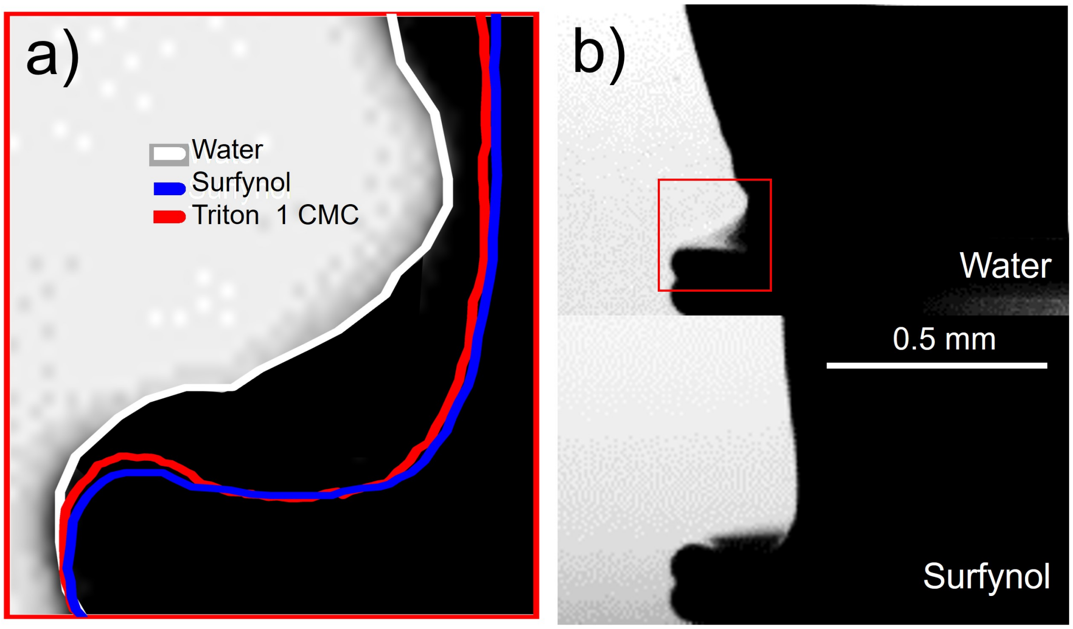

FIG. S2. Profile of the droplet lamella 0.5 milliseconds after impact at $U_0 = (1.20 \pm 0.06)\,\mathrm{m\,s^{-1}}$. a) Here, the water droplet is visible in the background. b) Droplet profiles in the vicinity of the contact line for Water & Surfynol.

time for each frame. The initial frame is identified as 0.0 ms, and consecutive frames are separated by the frame rate specified in the camera settings.

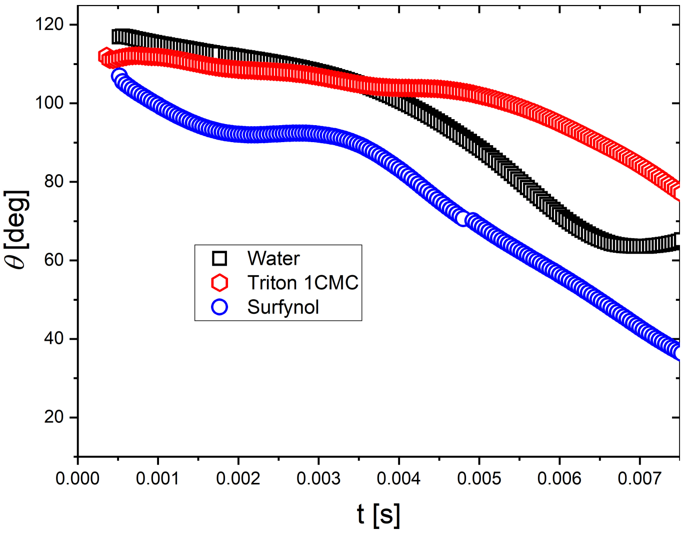

FIG. S3. Dynamic contact angle in terms of time from impact. The contact angle for Surfynol is below water at all times. In contrast, for Triton 1 CMC the contact angle is first below water but above after 7.0 ms. The impacted surface is polystyrene.

Contact line dynamics for all solutions

To demonstrate the effect of surfactants on droplet impact on the polystyrene and Teflon surfaces, we used six different solutions. In particular, we measured the dynamic contact angle and spreading diameter for each solution to characterise the influence of surfactants at the onset of impact. Figure 3 in the main text shows the dynamic contact angle and normalised spreading diameter for three liquids that represent extreme cases of surfactant action

and speed, only for the polystyrene surface. Figures S3 and S4 supplements Figure 3 by showing the dynamic contact angle for all six surfactant solutions on Teflon and polystyrene surfaces, respectively. The impact speed of all droplets was $U_0 = (1.00 \pm 0.02)\,\mathrm{m\,s^{-1}}$ for Teflon and $U_0 = (0.88 \pm 0.02)\,\mathrm{m\,s^{-1}}$ for polystyrene.

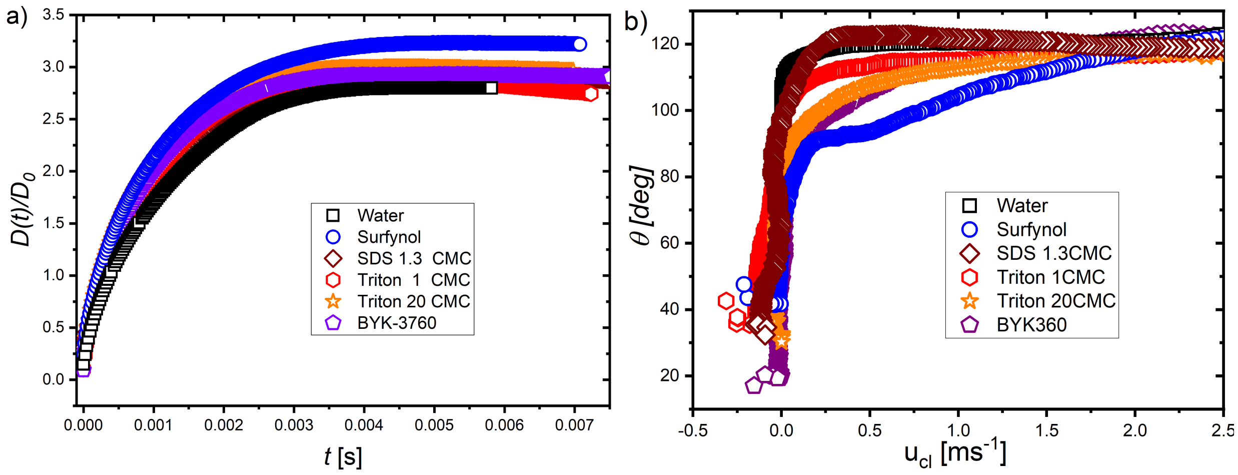

FIG. S4. Spreading dynamics following droplet impact on *Teflon* for all the liquids. a) Evolution of the spreading diameter $D(t)/D_0$ in terms of the time from impact. b) The dynamic contact angle θ_D in terms of the contact line velocity (u_{cl}). The impact velocity for these experiments is $U_0 = (1.00 \pm 0.02)\,\mathrm{m\,s^{-1}}$.

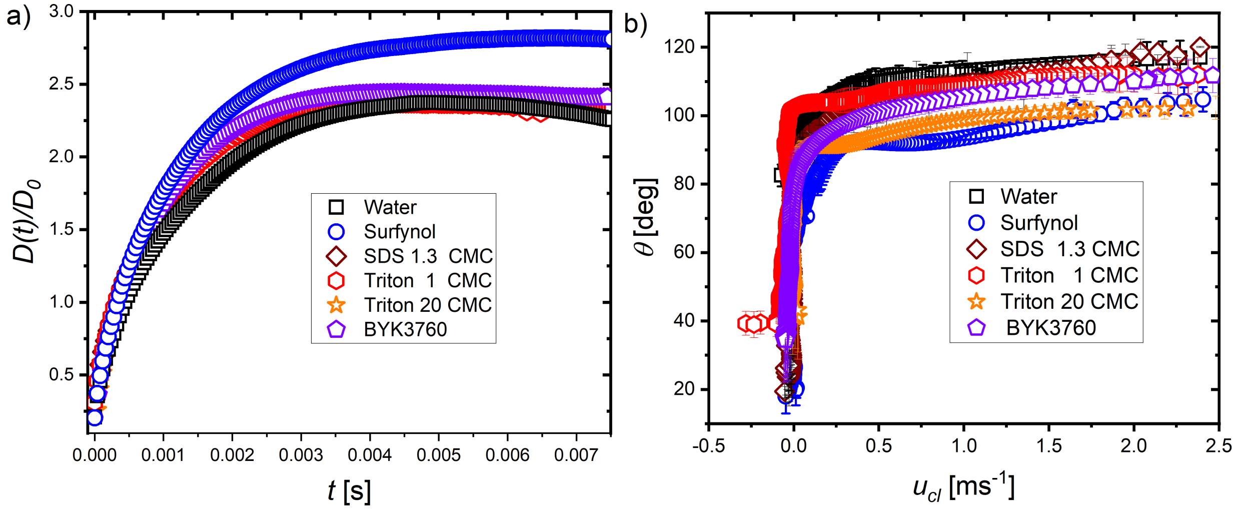

FIG. S5. Spreading dynamics following droplet impact on *polystyrene* for all the liquids. a) Evolution of the spreading diameter $D(t)/D_0$ in terms of the time from impact. b) The dynamic contact angle θ_D in terms of the contact line velocity (u_{cl}). The impact velocity for these experiments is $U_0 = (0.88 \pm 0.02)\,\mathrm{m\,s^{-1}}$.

Maximum Spreading

Attempts at investigating the maximum spreading factor were carried out and compared with recent models. However, it is important to highlight that these models *do not incorporate the effects of varying surface tension* (temporally and spatially), or surfactant distribution. These plots, therefore, simply represent an attempt at exploring the compatibility between our data and recent models. What makes the use of these models for comparison less accurate when studying surfactant-laden flows is the need of a fixed value for the surface tension. Here, the value of the surface tension used in these calculations corresponds to the value obtained with our tensiometer at a bubble

lifetime (surface age) equal to the time at which the drop reaches its maximum diameter. We note, however, that as presented elsewhere (Huet, O. D. Y. *et al* 2024, arXiv preprint arXiv:2310.13288), and our own investigations indicate, the actual value of the surface tension when surfactants are presents may be different overall and at different locations throughout the surface of the droplet and the contact line, and at different time-scales during the spreading process - an effect that seems to become more prominent at higher impact speeds.

For Figure S6a), the maximum spreading d_m of the liquids was computed following the Padé approximant based on the findings by Laan et al. in 2014 (Phys. Rev. Applied, **2** 044018, 2014). The plot shows agreement within error bars for the impacts on polystyrene, with the results for Teflon being one standard error away. We note Laan's model neither considers the influence of the substrate wettability nor dynamic surface tension effects, so this could be the reason of the discrepancy.

Aksoy et al. in 2022 (Phys. Fluids, **34** 4, 2022) proposed a model for the maximum spreading diameter that incorporates the influence of viscous dissipation and wetting characteristics, but similar to the model by Laan et al, it was developed for surfactant-free drops. Therefore, the dynamic effects of the surface tension and surfactant distribution are not taken into account. The derivation of the model involves an energy balance, where the viscous dissipation term was based on existing data, leading to the maximum spreading diameter to be modelled as:

$$3.18\frac{We^{0.72}}{Re^{0.86}}d_m^{6.5} + 3(1 - cos\theta)d_m^3 - (We + 12)d_m + 8 = 0. \tag{S1}$$

We evaluated the performance of the Aksoy et al. model by solving Equation S1 using the values of We and Re obtained from our experimental measurements, with the contact angle θ taken at the maximum spreading diameter. In Figure S6b), we show these results with different grey bands representing how far, in percentile error, the processed data is from the prediction (for instance, 10 % for the Teflon data, while 30% for polystyrene).

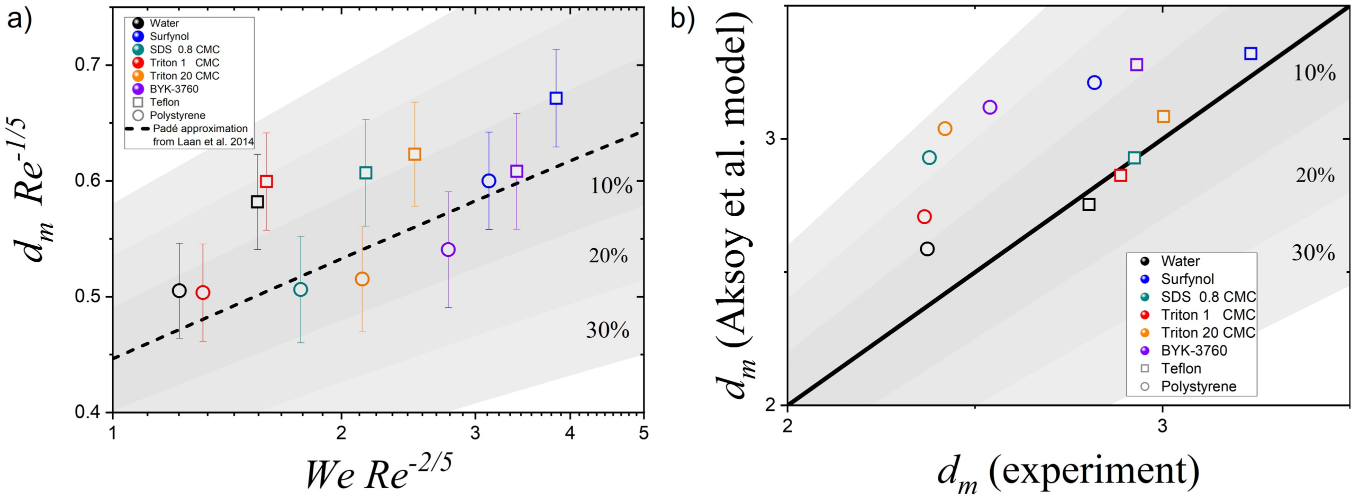

FIG. S6. a) Maximum spreading factor in terms of $P = \mathrm{WeRe}^{-2/5}$.The experiments in polystyrene and Teflon were performed at impact speeds $U_0 = 0.88 \pm 0.01$ ms^{-1}, and $U_0 = 1.01 \pm 0.01$ ms^{-1}. In addition, the dashed line shows the Padé approximant $d_m\mathrm{Re}^{-1/5} = \frac{P^{1/2}}{1.24+P^{1/2}}$. b) Experimental data assessed against the Aksoy et al. model from equation 1. The aid visualisation, error bands in grey scales from 10% to 30% are shown.
